# Accurate quantification of pulmonary perfusion ratio in children with congenital heart disease using partial volume corrected 4D flow cardiac magnetic resonance

**DOI:** 10.3389/fped.2024.1339679

**Published:** 2024-05-16

**Authors:** Kerstin Lagerstrand, Anna Nyström, Pär-Arne Svensson, Charlotte De Lange, Frida Dangardt

**Affiliations:** ^1^ Department of Medical Physics and Biomedical Engineering, Region Västra Götaland, Sahlgrenska University Hospital, Gothenburg, Sweden; ^2^ Institute of Clinical Sciences, Sahlgrenska Academy, University of Gothenburg, Gothenburg, Sweden; ^3^ Department of Pediatric Radiology, Region Västra Götaland, Sahlgrenska University Hospital, Gothenburg, Sweden; ^4^ Children's Heart Center, The Queen Silvia Children's Hospital, Region Västra Götaland, Sahlgrenska University Hospital, Gothenburg, Sweden; ^5^ Institute of Medicine, Sahlgrenska Academy, University of Gothenburg, Gothenburg, Sweden

**Keywords:** MRI, scintigraphy, partial volume correction, lung perfusion, congenital heart disease

## Abstract

**Background:**

In children with congenital heart disease (CHD), lung scintigraphy is the reference standard for evaluation of pulmonary perfusion. 4D flow CMR offers a non-ionizing alternative. Due to the intrinsic limitation in the spatial resolution, however, 4D flow may display clinically unacceptable differences compared to the reference standard. This case study aims to highlight the importance of correcting for such partial volume errors to accurately evaluate pulmonary perfusion in small pulmonary arteries.

**Methods:**

Children with CHD, mainly those with transposition of the great arteries or tetralogy-of-Fallot, referred to CMR from 2020 to 2022 at our clinic, were retrospectively reviewed; *n* = 37. All patients had been examined with a free breathing, motion-corrected 4D flow protocol. Comparison in pulmonary perfusion (PPR: relative flow through right and left pulmonary arteries) with scintigraphy were performed both for 4D flow before and after partial volume correction.

**Results:**

Patients with large pulmonary arteries, 76%, displayed small differences in PPR between modalities (<20%), while patients with arteries of only a few pixels, 24%, displayed differences up to 178%, depending on the relative difference in size between the right and left pulmonary artery. Differences were effectively reduced after partial volume correction (<21%).

**Conclusion:**

The present report shows that 4D flow is a promising tool to accurately evaluate the pulmonary perfusion in children with CHD, but that partial volume correction is warranted to overcome its limitation in the spatial resolution. Without such correction, lung scintigraphy is still recommended to ensure high diagnostic certainty in children with small pulmonary arteries.

## Introduction

Pulmonary blood flow is often reduced in patients with congenital heart defects (CHD). In patients with transposition of the great arteries (TGA) or tetralogy-of-Fallot (TOF), decreased pulmonary blood flow may affect the development of the lungs and the right heart, leading to poorer outcome (1–5). TGA is a rare but serious heart defect, in which the two main arteries leaving the heart are transposed, resulting in two separate circulations that do not exchange oxygen efficiently. This abnormal morphology requires surgical intervention for correction, typically through the arterial switch procedure, e.g., the Rastelli procedure or the Nikaïdoh technique (5). The surgical correction could alter the pulmonary blood flow distribution and lead to complications such as pulmonary hypertension and right ventricular dysfunction. Many patients with TOF have residual right ventricle outflow obstruction that can lead to decreased pulmonary blood flow and increased resistance in the pulmonary circulation. The abnormal morphology can be surgically corrected with a transannular patch or pulmonary homograft (1–5). After surgery, pulmonary branch stenosis is not uncommon in both TOF and TGA. The concomitant elevated and increasing pulmonary vascular resistance may cause reduced exercise capacity as well as heart failure (4, 6).

Due to advancements in medical care and improvements in patient selection and perioperative management, the survival rate for patients with TGA and TOF has improved significantly over time (1–5). Ongoing advancements in postoperative care, including improved monitoring and follow-up protocols, have contributed to better outcomes and long-term survival. Imaging is often used in the clinical setting to detect possible morphological and functional changes and plan the best treatment for each unique patient. At present, lung perfusion scintigraphy, which measures the perfusion in the entirety of the lungs, is the reference standard to evaluate pulmonary perfusion in patients with CHDs (7, 8). Non-ionizing diagnostic tools are warranted to reduce radiation exposure in this young population requiring lifetime surveillance. With the introduction of 4D flow cardiovascular magnetic resonance (CMR) in the examination protocol, the pulmonary blood flow distribution can be determined without harmful ionization and in any desired plane. Due to the intrinsic limitation in the spatial resolution in relation to the true vessel size (9–11), however, the 4D flow CMR may display clinically unacceptable differences compared to the reference standard.

The present report aims to highlight the importance of correcting for these so-called partial volume effects to accurately evaluate pulmonary perfusion using 4D flow CMR.

## Methods

### Study population

Children with CHDs, mainly those with TGA and TOF, referred to CMR from 2020 through 2022 at our clinic, were retrospectively reviewed (Ethical approval Dnr 802-12 and 2022-01131-02). Patients with collateral circulation such as aortopulmonary collaterals or other shunting to the lungs were excluded.

### Examination protocol

#### CMR examination

All patients had been examined without sedation on a 3T MRI scanner (Signa, GE Medical Systems, Waukesha, WI, USA) using an AIR technology anterior array with 20–30 channels in 3 different sizes. The anterior array was combined with the standard posterior coil, integrated in the tabletop. In addition to conventional clinical sequences, a free breathing, motion compensated retrospectively ECG gated 4D flow protocol was included (TR: 4.0–4.4 ms; TE: 2.2–2.3 ms; flip angle: 15°; receiver bandwidth: 31.25 kHz; field-of-view: 360–440 × 360–440 mm^2^; acquisition voxel size: 2 × 2 × 2–2.2 × 2.2 × 2.2 mm^3^; number of excitations: 4, hyperkat acceleration = 6–8× and temporal resolution 31–63 ms). The examination was performed in free breathing with respiratory compensation between 10% and 20% (% *k*-space compensation). The velocity encoding was chosen to be 20% lower than highest expected flow velocity, i.e., 80–400 cm/s. To enhance the blood contrast and facilitate vessel delineation in the 4D flow images, 0.1 mmol/kg body weight Gadoterate meglumine contrast media was injected before the 4D flow CMR acquisition. The full CMR examination was completed within 45–60 min, whereas the 4D flow CMR acquisition lasted for 5–10 min.

**Figure 1:**
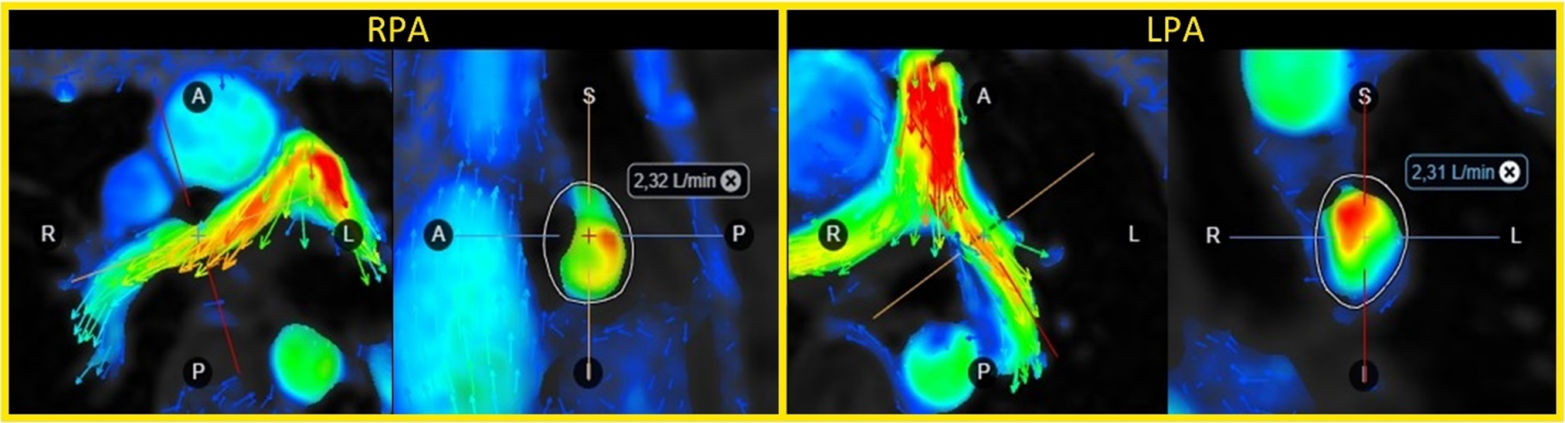
Flow volume evaluation in the right (RPA) and left pulmonary artery (LPA) using 4D flow CMR in a 16-year patient with tetralogy-of-Fallot and a transannular patch.

The 4D flow data was post-processed offline using the commercially available software Cardio AI (Arterys, Redwood Shores, USA). A senior CMR radiologist (A.N.), blinded to the results, determined the optimal position in the velocity images of the right and left pulmonary arteries for evaluation of the lung perfusion. The velocity images were, then, carefully adjusted and reconstructed in a double-oblique plane perpendicular to the dominant flow direction. From these velocity images, the volume of blood flows into the lungs were calculated as the net flow volume passing through the right and left pulmonary arteries during the cardiac cycle (Figure 1). Finally, the pulmonary perfusion ratio (PPR) was determined as the right flow volume divided by the left flow volume.

#### Partial volume correction

For small vessels with less than 3.5 voxels over the vessel diameter, large errors in the volume of blood flow may occur due to partial volume effects (9). For partial volume correction, a previously proposed method that estimates the corrected flow volume, *Q*_corr_, from the acquisition pixel size, Δx, as well as the measured vessel radius, *r*_meas_ and flow volume, *Q*_meas_, for was used (11) according to Equation 1:

(1) $$Q_{\mathrm{corr}}=Q_{\mathrm{meas}}\left[-0.2\left(\frac{\Delta x}{r_{\mathrm{meas}}}\right)+1\right]$$

#### Lung perfusion scintigraphy

In the included cohort, patients who had undergone lung scintigraphy as part of their routine clinical follow-up were identified. The perfusion distribution measurements in these patients were then used as a standard reference for comparison with the 4D flow measurements. The lung scintigraphy was performed after intravenous administration of 1 MBq/kg body weight ^99m^Tc-labeled human albumin macroaggregates (MAA), with 5,000 particles/MBq (particle size 20–100 μm) according to the guidelines of the European Association of Nuclear Medicine (12). As such, the image acquisition started within minutes of the administration. The patient was imaged in supine position directly on the collimator, using a dual-head gamma camera (GE) with a low-energy, high-resolution collimator. Static perfusion images were obtained in 256 × 256 matrix format with 200–500 *k* counts per view, zoomed to include both lungs within the field-of-view. Anterior, posterior, left and right anterior and posterior oblique views were collected. The lung perfusion ratio was calculated as the geometric mean of the counts from both lungs in the anterior and posterior views. The lung scintigraphy examination was approximately 15 min.

### Descriptive and inferential statistics

All statistical analyses were performed using the SPSS Statistics software (IBM Corp. Released 2021. IBM SPSS Statistics for Windows, Version 28.0. Armonk, NY: IBM Corp).

For all included patients, the difference in PPR between the modalities before and after partial volume correction of the 4D flow measurements was plotted against the vessel size to visualize the dependence of the PPR estimate on the vascular dimension. For those patients who had undergone lung perfusion scintigraphy, the difference in PPR between the modalities was determined with and without partial volume correction. Possible differences in PPR between the modalities were further highlighted by Bland-Altman analysis. Additionally, the Pearson correlation coefficient was used to determine correlation between modalities.

## Results

During the 2 years of inclusion, 37 children with TOF and TGA had been examined with CMR, including 4D flow CMR at our clinic. The included patients spanned over a large age range and body surface areas (Table 1). The majority of children had repaired TOF (*n* = 23), 12 patients had TGA and one patient had tricuspid atresia and pulmonary artery stenosis. Nine of the patients had CHD repaired with an arterial switch operation (ASO), 17 with a transannular patch (TAP), 4 with a pulmonary homograft, one had a Nikaïdoh operation and one had undergone the Rastelli procedure (Supplementary Table S1). A few patients had MR compatible implants but with a placement not interfering with 4D flow measurements.

**Table 1:** Baseline patient characteristics.

Parameter	mean ± SD
Age (years)	12.2 ± 4.5
Males (%)	57
Height (cm)	149.2 ± 27.1
Weight (kg)	47.2 ± 19.6
BSA	1.4 ± 0.4

BSA, body surface area; SD, standard deviation.

**Figure 2:**
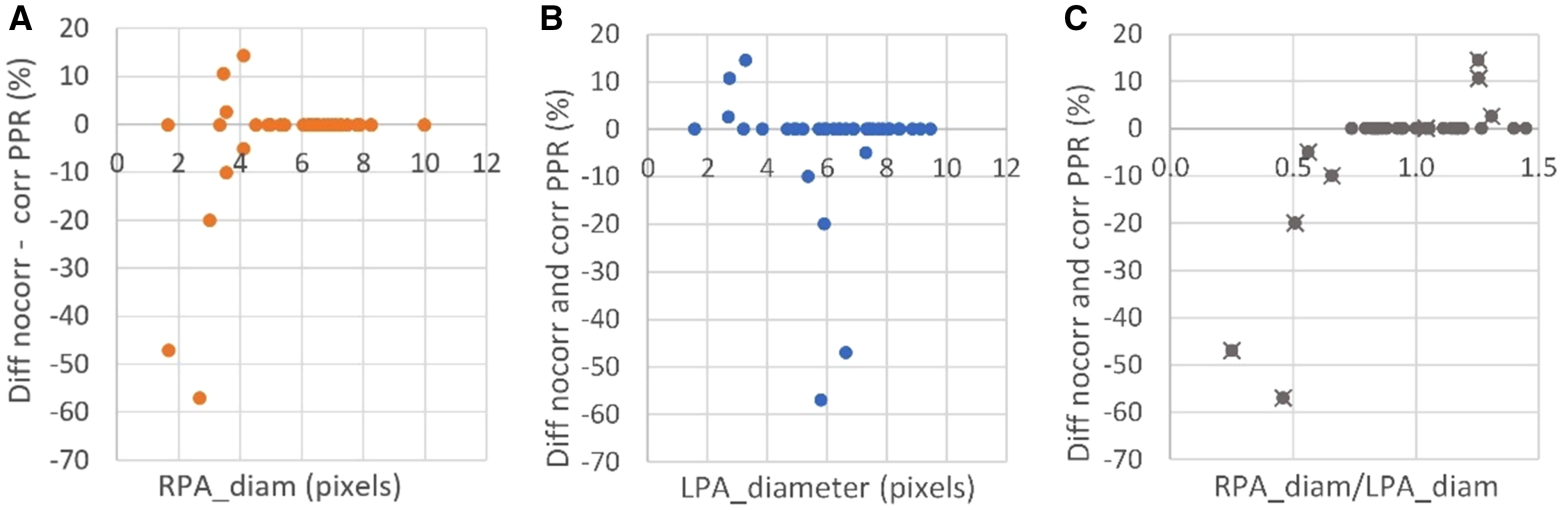
The difference in pulmonary perfusion ratio (PPR) with and without partial volume corrected 4D flow CMR vs. (**A**) the right pulmonary artery (RPA) and (**B**) left pulmonary artery (LPA) diameter in all included patients, as well as the PPR difference vs. (**C**) the relative diameter (patients that had at least one small pulmonary artery with <3.5 voxels over the vessel diameter are indicated with a cross).

Of all patients included, 76% were found to have sufficiently large pulmonary arteries without limitation in the spatial resolution of the 4D flow measurement, i.e., pulmonary arteries with size >3.5 voxels over the vessel diameter. Of the patients with TOF, only one (4%) had small pulmonary arteries with size <3.5 voxels over the vessel diameter and, as such, was in need of partial volume correction. In the TGA cohort, the number of patients with smaller pulmonary arteries was approximately ten times higher (43%). The misestimation of the 4D flow based PPR due to the partial volume effect is shown in Figure 2, as the difference between corrected and non-corrected data. As shown in the figure, the misestimation was found to depend not only on the vessel size but also on the relative difference in size between the right and left pulmonary arteries. 4D flow CMR overestimated PPR when the left pulmonary artery was larger than the right and underestimated the PPR when the right pulmonary artery was larger than the left. In patients with bilaterally small arteries, however, the calculation of the PPR quota neutralized the partial volume error.

**Figure 3:**
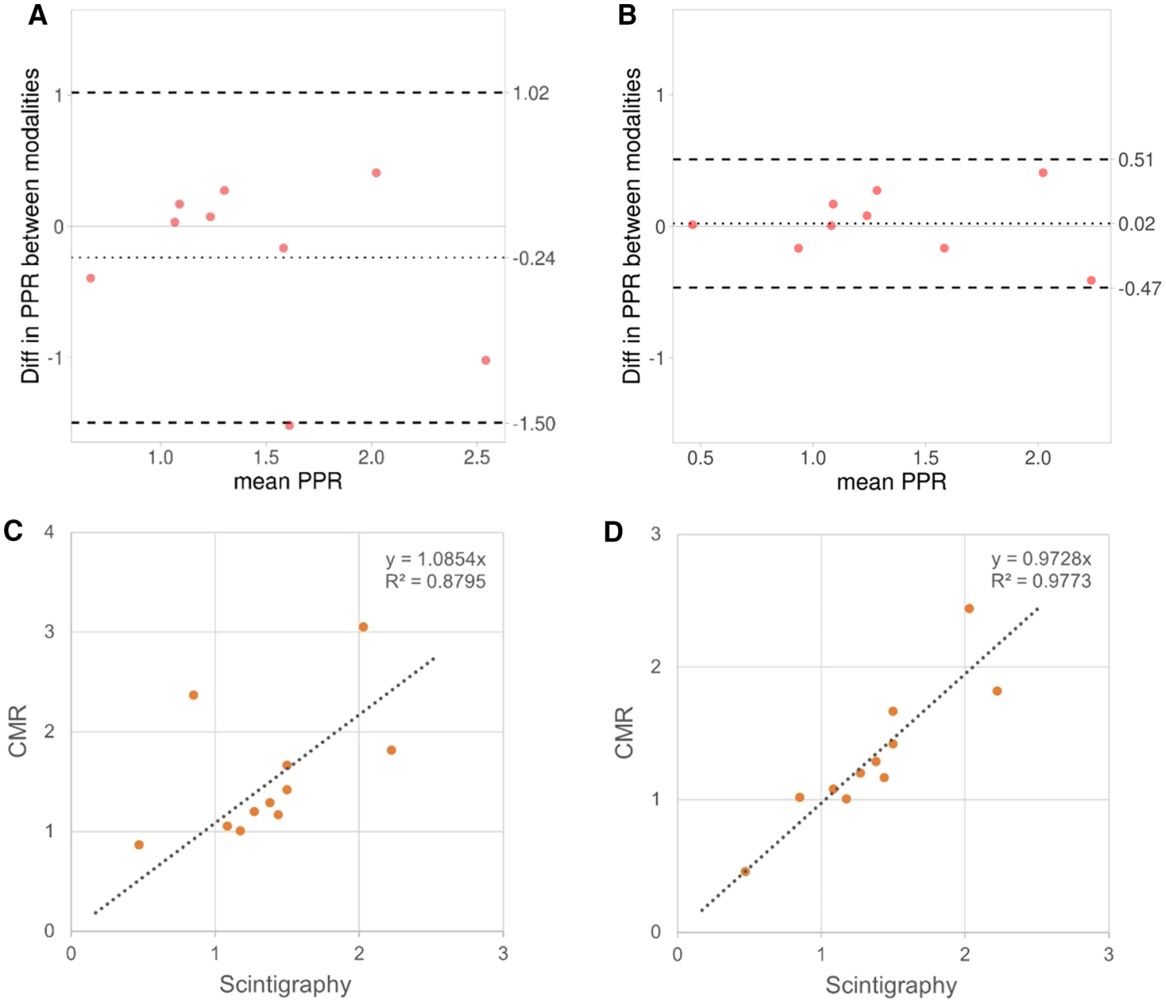
In the eleven patients that had undergone examinations both with lung scintigraphy and 4D flow CMR, the bland Altman plots displayed larger bias and wider limits of agreement in pulmonary perfusion ratio (PPR) between lung scintigraphy and 4D flow CMR before (**A**) than after partial volume correction (**B**). The corresponding Pearson correlation QQ-plots (**C** and **D**, respectively) displayed increased linearity between the modalities after correction.

Eleven patients had been characterized with lung scintigraphy before CMR as part of their routine clinical follow-up. The time interval between CMR and scintigraphy was on average 2,618 (SD: 1,295) days. No surgical procedures, or interventions were performed between the scintigraphy and CMR examinations. As can be seen from Table 2, small differences in PPR between 4D flow CMR and lung scintigraphy were found for patients with large pulmonary arteries (*n* = 7; difference: <20%). However, the four patients with smaller pulmonary arteries displayed larger differences in PPR between the modalities (max difference: 178%); this difference was effectively reduced after partial volume correction to less than 21%. After correction, the correlation in PPR between the modalities increased (before: *R* = 0.94; *p* < 0.001; after correction: *R* = 0.99; *p* < 0.001) and the Bland Altman plots clearly displayed, with smaller bias [difference: −0.24 (−0.76, 0.28) vs. 0.02 (−0.18, 0.22)] and smaller limits of agreement [upper limit of agreement: 1.02 (0.12, 1.91) vs. 0.51 (0.16, 0.86); lower: −1.5 (−2.39, −0.60) vs. −0.47 (−0.81, −0.12)], the value of the partial volume correction on the evaluation of pulmonary function using 4D flow CMR (Figure 3).

**Table 2:** Difference in the estimated pulmonary perfusion between lung scintigraphy and 4D flow CMR in the 11 patients that had undergone examinations with both modalities. For patients with small vessels, in need of partial volume correction, the estimated pulmonary perfusion after partial volume correction is also reported. Patient demographics are presented in the Supplementary Table S1.

ID	RPA_diam_		LPA_diam_		RPA RF	LPA RF	RPA_diam_	RPA_flow_		LPA_flow_		Scint	Scint	PPR_scint_	PPR_PCCMR_		ΔPPR_scint-PCCMR_	
							/LPAdiam	no corr	corr	no corr	corr	right	left		no corr	corr	no corr	corr
	mm	pix	mm	pix	%	%		L/min	L/min	L/min	L/min	%	%				%	%
1	5.0	1.7	19.9	6.6	18	84	0.3	1.3	0.7	1.5	1.5	32	68	0.5	0.87	0.46	−84.2	2.4
2	8.0	2.7	17.4	5.8	43	36	0.5	3.4	1.5	1.4	1.4	46	54	0.9	2.37	1.02	178.3	19.7
3	9.0	3.0	17.7	5.9	25	21	0.5	8.7	7.0	2.9	2.9	67	33	2.0	3.05	2.44	−50.4	20.3
4	21.8	7.3	26.6	8.9	23	41	0.8	5.0	–	3.0	–	60	40	1.5	1.67	–	−11.1	–
5	20.1	6.7	24.2	8.1	5	33	0.8	2.4	–	2.0	–	56	44	1.3	1.20	–	5.7	–
6	14.7	4.9	15.6	5.2	32	17	0.9	28.7	–	28.5	–	54	46	1.2	1.01	–	14.3	–
7	16.3	5.4	17.2	5.7	40	15	0.9	2.0	–	1.1	–	69	31	2.2	1.82	–	18.3	–
8	18.9	6.3	17.9	6.0	1	4	1.1	2.7	–	1.9	–	60	40	1.5	1.42	–	5.3	–
9	29.9	10.0	23.6	7.9	5	3	1.3	2.8	–	2.4	–	59	41	1.4	1.17	–	18.9	–
10	10.6	3.5	8.1	2.7	0	15	1.3	14.2	11.4	13.5	10.5	52	48	1.1	1.05	1.08	2.9	0.4
11	19.6	6.5	14.0	4.7	13	17	1.4	61.5	–	47.7	–	58	42	1.4	1.29	–	6.6	–

RPA, right pulmonary artery; LPA, left pulmonary artery; PPR, pulmonary perfusion ratio; PC, phase contrast; CMR, cardiac magnetic resonance.

## Discussion

Reliable quantification and distribution of pulmonary perfusion is of clinical importance in the peri- and postoperative period, as well as in the long-term surveillance of many types of CHD. 4D flow CMR, which can provide detailed information about the blood flow dynamics in the cardiovascular tree, has been pointed out as a promising non-ionizing tool for the evaluation of pulmonary perfusion (13). This case study highlights the value of using partial volume corrected 4D flow CMR for such evaluations. Without addressing the intrinsically limited spatial resolution of the technique, the perfusion evaluation will be afflicted with significant errors that may have an impact on the medical judgment and decision-making of treatment in a clinical context.

When monitoring possible complications associated with CHDs, full workup-plans and comprehensive diagnostic assessments are often required. One of the advantages of CMR is the versatility of the modality. Besides evaluating the blood hemodynamics by 4D flow, the vascular anatomy can be imaged with high spatial detail by CMR angiography. Moreover, ventricular function and changes in ventricular volumes can be detected by CINE CMR. Such information is crucial because right ventricular end-diastolic volume, for example, is a strong predictor of death and transplant-free survival (14).

The pulmonary vasculature increases in size and changes naturally over time due to aging, but in patients with TGA or TOF, the pulmonary vasculature is subjected to abnormal physiological changes that most likely affect the vasculature growth. As the result of the alterations in the pulmonary vasculature, the pulmonary perfusion may also undergo changes, and this can lead to true reductions in pulmonary blood flow and even to complications such as pulmonary hypertension and right ventricular dysfunction (3, 5). Hence, these patient cohorts need close surveillance with repeated monitoring over the course of their lifetimes, starting in childhood. Lung scintigraphy is the reference standard for surveillance of pulmonary perfusion in patients with CHD (7, 8), but 4D flow is an attractive alternative. Compared with lung perfusion scintigraphy, 4D flow CMR offers several advantages. Firstly, the technique does not rely on the injection of radioactive tracers and, as such, does not expose sensitive lung tissue to harmful ionizing radiation. This is especially important in children, who are more sensitive to radiation and have a higher risk of developing cancer (15). With 4D flow CMR, detailed characterization of the ventricular volume and function and flow hemodynamics are also allowed within the same examination. On the other hand, regional pulmonary perfusion (on a segmental level) cannot for the moment be fully estimated with 4D flow CMR, as compared to lung perfusion scintigraphy. CMR is also contraindicated in individuals with specific pacemakers and leads, and for other implants, such as stents. When these devices are CMR compatible, the image quality may still be hampered, and scintigraphy is required. Additionally, as shown here by present findings, the measured pulmonary perfusion ratio can be highly overestimated or underestimated due to partial volume errors when the vessel size is small relative to the spatial resolution of the acquisition. The partial volume error increases with reduced vessel size and the effect on the perfusion ratio depends on the relative difference in the size of the right and left pulmonary arteries. This is an undesirable feature of a diagnostic tool that is used for surveillance and decision-making. Important clinical changes in pulmonary perfusion, which may occur over time, could then remain unnoticed if concomitant changes in the vasculature also occur. That is, changes in the vessel dimension may introduce partial volume errors in the measurements that counteract true changes in the pulmonary perfusion. This further underscores the significance of correcting for potential partial volume errors when performing 4D flow CMR.

4D flow CMR has been used as a research tool for several decades but has just recently found its way into clinical practice. This is partly due to the substantial reduction of the scan and post-processing time as compared to multiple two-dimensional through-plane flow CMR measurements. Also, with 4D flow CMR, information can be collected from a volume of the body, where large coverage offers the possibility to retrospectively choose an arbitrary site for the measurement of blood flow. This enables repositioning and optimization of measurements without additional scan time. However, the slightly lower spatial resolution of the technique in comparison to conventional two-dimensional through-plane flow CMR may further limit the pulmonary perfusion evaluation in patients with small vessels by introducing larger errors in the estimate if not corrected for. 4D flow CMR was implemented 4 years ago in our hospital for assessment and management of children with CHD, but also to assist in surgical planning and evaluate postoperative complications. In our experience and as shown here, 4D flow CMR offers reliable quantification of lung perfusion ratio in most patients that is comparable to lung perfusion scintigraphy, and has, thereby, potential to contribute to improved patient care and outcomes in children with conditions like TGA and TOF. However, we found that a few of the TOF patients and almost 50% of the TGA patients had small pulmonary arteries with diameter <10.6 mm (i.e., <3.5 voxels over the vessel diameter) and, therefore, runs the risk of being misdiagnosed with 4D flow CMR. These patients can be readily identified in the clinical setting based on the size of the vessel, and then appropriate corrections could be made using for example the proposed method.

## Study limitations

Some limitations in the study design should be mentioned. Consistent with the single-center study design and the low incidence of CHDs, the sample size was rather small during the period of inclusion. However, the study was not designed as a group comparison study but a case cohort study that aimed to highlight the value of partial volume corrected 4D flow CMR for pulmonary perfusion evaluation and displaying the size of the error and the percentage of patients in the clinic that may be misdiagnosed without such correction. Furthermore, the time between the scintigraphy and CMR was long, and the lung scintigraphy measurements may have been afflicted by methodological errors that may explain the remaining bias between the modalities. Prefeential drainage of the caval blood into the right or left pulmonary arteries may also introduce differences between CMR and lung scintigraphy in patients with Fontan circulation (16). To reduce such influence on the results, the present study only included patients without caval connection to the pulmonary arteries.

## Conclusions

With the implementation of 4D flow CMR as a comprehensive diagnostic tool in workup plans, CHD patients can take advantage of the benefits of the CMR technology, providing versatile imaging capabilities, allowing for longitudinal monitoring, and facilitating early detection of complications. Present findings clearly highlight the value of partial volume correction for 4D flow-based evaluation of pulmonary perfusion in patients with CHD. With such correction, CMR could be used also for surveillance of patients with small pulmonary arteries, reducing the radiation burden associated with lung perfusion scintigraphy. This is of particular interest for pediatric healthcare. Even though the partial volume effect is a well-known source of error in phase contrast flow techniques, which 4D flow CMR is based upon, commercial analysis tools currently do not incorporate algorithms for partial volume correction. Present work underscores the need for such actions.

## Data Availability

The original contributions presented in the study are included in the article/**Supplementary Material**, further inquiries can be directed to the corresponding author. Requests to access the datasets should be directed to xlagke@gu.se.
