# Supplementary material for: Accurate quantification of pulmonary perfusion ratio in children with congenital heart disease using partial volume corrected 4D flow cardiac magnetic resonance
Source: Front Pediatr. 2024 May 16;12:1339679. doi: 10.3389/fped.2024.1339679 (PMC11137306; doi:10.3389/fped.2024.1339679)
Supplement: Supplementary file 1 [file Table1.pdf]

Supplementary Table 1. Patient demographics.

| ID | Umbrella diagnosis | Diagnosis | Current clinical problem | sex | height | weight | BSA | age |
|---|---|---|---|---|---|---|---|---|
| | | | | male=1 | cm | kg | | year |
| 1 | TOF | TAP | PI + RPA stenosis | 1 | 135 | 41 | 1.2 | 9 |
| 2 | TA | IAA | Homograft + RPA stenosis | 0 | 155 | 45 | 1.4 | 12 |
| 3 | TGA | VSD, ASO | Status post-ASO | 0 | 172 | 60 | 1.7 | 18 |
| 4 | TOF | TAP | PI | 0 | 167 | 51 | 1.6 | 13 |
| 5 | TOF | TAP | PI | 0 | 164 | 58 | 1.6 | 18 |
| 6 | PA | VSD, PA-conduit | PI | 1 | 176 | 60 | 1.7 | 16 |
| 7 | TOF | TAP | PI | 1 | 153 | 44 | 1.4 | 11 |
| 8 | TOF | 22q11, TAP | PI | 1 | 147 | 65 | 1.6 | 12 |
| 9 | TOF | TAP | PI | 0 | 159 | 92 | 1.9 | 17 |
| 10 | TGA | DORV, CoA, ASO, AI | Status post-ASO, AI | 1 | 111 | 18 | 0.8 | 7 |
| 11 | TGA | ASO | Status post-ASO, AI | 1 | 183 | 93 | 2.2 | 17 |
| 12 | TGA | VSD, PA-conduit | Status post-op Nikaidoh | 1 | 176 | 75 | 1.9 | 15 |
| 13 | TOF | TAP | PI | 0 | 129 | 23 | 0.9 | 9 |
| 14 | TOF | TAP | PI | 0 | 162 | 66 | 1.7 | 13 |
| 15 | TOF | PI | PI | 1 | 172 | 62 | 1.7 | 18 |
| 16 | TOF | TAP | PI | 0 | 164 | 48 | 1.5 | 16 |
| 17 | TOF | TAP | PI | 1 | 180 | 60 | 1.8 | 15 |
| 18 | TOF | TAP | PI | 1 | 169 | 48 | 1.5 | 17 |
| 19 | TOF | TI | TI | 1 | 138 | 34 | 1.1 | 9 |
| 20 | TOF | PA-conduit | conduit stenosis | 1 | 152 | 55 | 1.5 | 17 |
| 21 | TGA | ASO | Status post-ASO | 1 | 183 | 56 | 1.7 | 16 |
| 22 | TGA | ASO | Status post-ASO | 1 | 157 | 49 | 1.5 | 12 |

| | | | | | | | | |
|---|---|---|---|---|---|---|---|---|
| 23 | TOF | TAP | PI | 1 | 139 | 29 | 1.1 | 10 |
| 24 | TOF | TAP | PI | 0 | 174 | 59 | 1.7 | 17 |
| 25 | TOF | TAP | PI | 0 | 137 | 32 | 1.1 | 10 |
| 26 | TGA | VSD, op Rastelli | Status post-op Rastelli, PI + PS | 0 | 141 | 31 | 1.1 | 10 |
| 27 | TGA | ASO, TAP, PI | Status post-ASO, PI | 0 | 139 | 35 | 1.2 | 11 |
| 28 | TGA | ASO | Status post-ASO | 1 | 59 | 5 | 0.3 | 0 |
| 29 | TOF | PI | PI | 1 | 135 | 51 | 1.3 | 10 |
| 30 | TOF | PI | PI | 0 | 162 | 60 | 1.6 | 15 |
| 31 | TOF | PI | PI | 1 | 163 | 47 | 1.5 | 14 |
| 32 | TOF | TAP | PI + LPA stenosis | 0 | 157 | 45 | 1.4 | 13 |
| 33 | TGA | ASO | Status post-ASO | 0 | 157 | 52 | 1.5 | 12 |
| 34 | TOF | homograft PA | PI | 0 | 107 | 17 | 0.7 | 6 |
| 35 | TGA | VSD, DORV, ASO | Status post-ASO + Rastelli | 1 | 85 | 13 | 0.6 | 2 |
| 36 | TGA | ASO | Status post-ASO, PI | 1 | 103 | 16 | 0.7 | 3 |
| 37 | TGA | ASO | Status post-ASO | 1 | 171 | 60 | 1.7 | 15 |

BSA: body surface area; TGA: transposition of the great vessels, TOF: tetralogy of Fallot, AI: aortic valve insufficiency, TI: tricuspid valve insufficiency, PI: pulmonary valve insufficiency, TA: tricuspid atresia, IAA: Interrupted aortic arch, TAP: transannular patch, ASO: arterial switch operation, VSD: ventricular septal defect, DORV: double outlet right ventricle, CoA: coarctation of the aortal, Rastelli/Nikaidoh op: alternative techniques for correction of TGA.
